# Ordered and Disordered Carboxylic Acid Monolayers on Calcite (104) and Muscovite (001) Surfaces

**DOI:** 10.1021/acs.jpcc.2c01157

**Published:** 2022-05-18

**Authors:** Sander J. T. Brugman, Paolo Accordini, Frank Megens, Jan-Joris Devogelaer, Elias Vlieg

**Affiliations:** Radboud University, Institute for Molecules and Materials, Heyendaalseweg 135, 6525AJ Nijmegen, The Netherlands

## Abstract

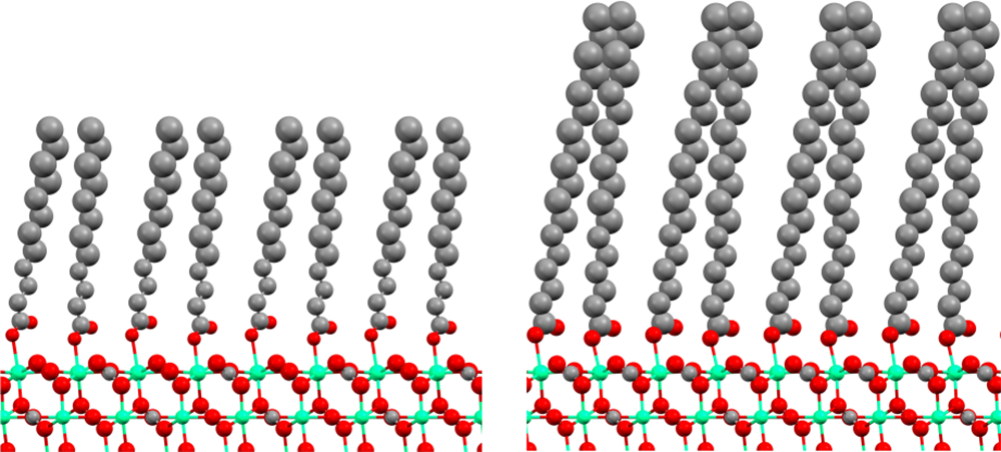

The adsorption of carboxylic acid molecules at the calcite (104) and the muscovite (001) surface was investigated using surface X-ray diffraction. All four investigated carboxylic acid molecules, hexanoic acid, octanoic acid, lauric acid, and stearic acid, were found to adsorb at the calcite surface. Whereas the shortest two carboxylic acid molecules, hexanoic acid and octanoic acid, showed limited ordering and a flexible, disordered chain, the two longest carboxylic acid molecules form fully ordered monolayers, i.e., these form highly structured self-assembled monolayers. The latter molecules are oriented almost fully upright, with a tilt of up to 10°. The oxygen atoms of the organic molecules are found at similar positions to those of water molecules at the calcite–water interface. This suggests that in both cases, the oxygen atoms compensate for the broken bonds at the calcite surface. Under the same experimental conditions, stearic acid does not adsorb to K^+^ and Ca^2+^-functionalized muscovite mica because the neutral molecules do not engage in the ionic bonds typical for the mica interface. These differences in adsorption behavior are characteristic for the differences of the oil–solid interactions in carbonate and sandstone reservoirs.

## Introduction

Interactions of organic molecules with clay and mineral surfaces are nowadays thought to be essential for the emergence of life on earth.^1^ These interactions also play an important role in many biogeochemical processes,^2^ in the transport of natural organic matter,^3^ in polymer materials,^4^ and in oil recovery.^5^ In oil recovery, the binding of oil components to minerals is generally stronger for carbonate than for sandstone reservoirs and therefore these reservoirs require different recovery conditions.^6,7^ Sandstone reservoirs contain predominately silicate minerals and clays. Muscovite mica, a phyllosilicate mineral, is a suitable model mineral for this type of reservoir because of its flatness^8^ and its similarity to clay minerals.^9^ Calcite (CaCO_3_) is a highly abundant mineral species in carbonate reservoirs and is also encountered in several organisms as a result of biomineralization.^10,11^ This makes calcite a favorite subject of study, also concerning the adsorption of organic molecules. It is well known that many organic molecules adsorb to the calcite surface, with long chain carboxylic acids or fatty acids having the strongest interactions.^12−14^ The atomic-scale calcite-adsorbate structure has been studied less. Hakim et al. used X-ray reflectivity to determine the structure of calcite in contact with the organic liquids methanol, isopropanol, pentanol, and octanoic acid.^15^ Earlier, Fenter and Sturchio used the same technique to determine the structure of the calcite–stearic acid interface in methanol and found adsorption of a stearic acid monolayer.^16^

In this work, we aim to compare the adsorption of carboxylic acid molecules on the basal plane of both calcite and muscovite mica. This yields an atomic-scale confirmation of the observations by RazaeiDoust et al. and provides therefore insight into the most important differences between sandstone and carbonate reservoirs.^7^ By including not only the specular rod as in X-ray reflectivity but also nonspecular rods, the three-dimensional interfacial structure can be determined. While a powerful structural tool, the interface needs to be very flat and homogeneous to apply surface X-ray diffraction and this limits the choice of organic molecules and minerals. The systems investigated here have therefore only partial relevance to the oil–mineral interfaces in actual reservoirs but do illustrate bonding options and provide detailed structural determinations. Systems found in nature typically contain water and ions that will affect the bonding and the interface structure,^17,18^ but also these will not be addressed here because we use methanol in order to dissolve the carboxylic acids. We have determined atomic positions of several carboxylic acids adsorbed on calcite and found that long chain length carboxylic acids form well-ordered monolayers. These monolayers do not only have a well-defined thickness, but they also exhibit ordering in the lateral direction. Carboxylic acids with a short chain do not show such strong ordering but do adsorb as flexible monolayers. This is in contrast to adsorption experiments on muscovite mica, which did not show adsorption of carboxylic acid molecules.

## Experimental Methods

### Sample Preparation

A calcite single crystal (Iceland spar, MTN Giethoorn) was freshly cleaved using a scalpel and hammer, exposing the (104) calcite surface plane. A 90% saturated solution was prepared by (1) equilibrating calcite crystals in water, (2) removing the calcite crystals, and (3) adding an additional volume of 10% water. Calcite was submerged in this 90% saturated solution for at least 1 h. This is expected to dissolve the crystal slightly, leading to a flat surface. The crystal was dried and subsequently placed in 100 mL of a 10 mM carboxylic acid solution in methanol. Hexanoic acid (Aldrich, ≥99.5% pure), octanoic acid (Fluorochem, 99% pure), lauric acid (Sigma, ≥99% pure), and stearic acid (Sigma-Aldrich, 95% pure) were used. After an equilibration time of 30 min in the carboxylic acid solution, the crystal was transferred into the surface X-ray diffraction (SXRD) cell, see Supporting Information S1. A few drops of the solution were added on top of the crystal to ensure a stable environment. Then, the crystal was covered by 13 μm-thick Mylar foil (Lebow Company) and mounted on the diffractometer. Excess liquid was removed by gently wiping over the Mylar foil using a tissue.

For stearic acid, also muscovite mica was used as a substrate. This material was freshly cleaved using a scalpel, resulting in a flat (001) surface.^8^ Muscovite mica pieces of approximately 45 × 45 mm^2^ were immersed in an aqueous solution containing 25 mM KCl (Sigma-Aldrich, ≥99.0% pure) or 25 mM CaCl_2_ (Merck, ≥99.5% pure).^19^ After more than 30 min in this solution, the sample was dried using a tissue and submerged in a 10 mM stearic acid solution in methanol, also for at least 30 min. The remainder of the sample preparation procedure was identical to that of calcite.

### Surface X-ray Diffraction

Surface X-ray diffraction (SXRD) measurements^20,21^ of stearic acid were conducted at the ID03 beamline of the European Synchrotron Radiation Facility (ESRF) on a *z* axis diffractometer with the crystal mounted horizontally and using a MAXIPIX area detector. For this experiment, a beam size of 325 × 29 μm^2^ and X-ray energy of 24 keV were used. All other surface X-ray diffraction measurements were performed at the I07 beamline of the Diamond Light Source, using a (2 + 2)-type diffractometer with the crystal mounted horizontally and a Pilatus 100 K area detector. A beam size of 200 × 20 μm^2^ and X-ray energy of 23 keV were used.

The calcite atomic positions and lattice parameters (*R*3̅*c*, *a* = *b* = 4.9900 Å, *c* = 17.061 Å, α = β = 90.0 and γ = 120.0° in the hexagonal setting) were obtained from Graf.^22^ The unit cell was transformed to have a surface unit cell parallel to the surface plane.^23,24^ This results in a surface unit cell with lattice parameters *a* = 8.0960 Å, *b* = 4.9900 Å, *c* = 24.2880 Å, α = 90.0°, β = 90.75°, and γ = 90.0°.^24^ The atomic positions of lauric acid were obtained from Bond.^25^ Hydrogen atoms were omitted because of the insensitivity of X-ray diffraction to these atoms. Lauric acid atomic positions were used to construct the stearic acid atomic positions by manually extending the molecule with six carbon atoms. The obtained structure was in reasonable agreement with some of the polymorphs of stearic acid.^26,27^ Similarly, the lauric acid atomic positions were used for the hexanoic acid and octanoic acid atomic positions, by shortening the chain to the appropriate length. Anomalous dispersion coefficients^28^ and atomic scattering factors^29^ were used. A constant angle of incidence of 1.2° was used for nonspecular crystal truncation rods. We did not observe changes in the measured crystal truncation rods over time, which indicates that the interface is not damaged by the X-ray beam. The agreement factor in the data was determined from symmetry equivalent reflections and lies between 5.0% and 16.6%. To increase the relative weight of surface-sensitive data points, data points close to a Bragg peak were given a larger error up to 30%. The “ARTS” Matlab script was used to convert integrated intensities into structure factors. The obtained structure factors were fitted with a model of the interfacial structure using the ROD software.^30^

## Results and Discussion

### Lauric Acid and Stearic Acid on Calcite

#### Crystal Truncation Rods

**Figure 1:**
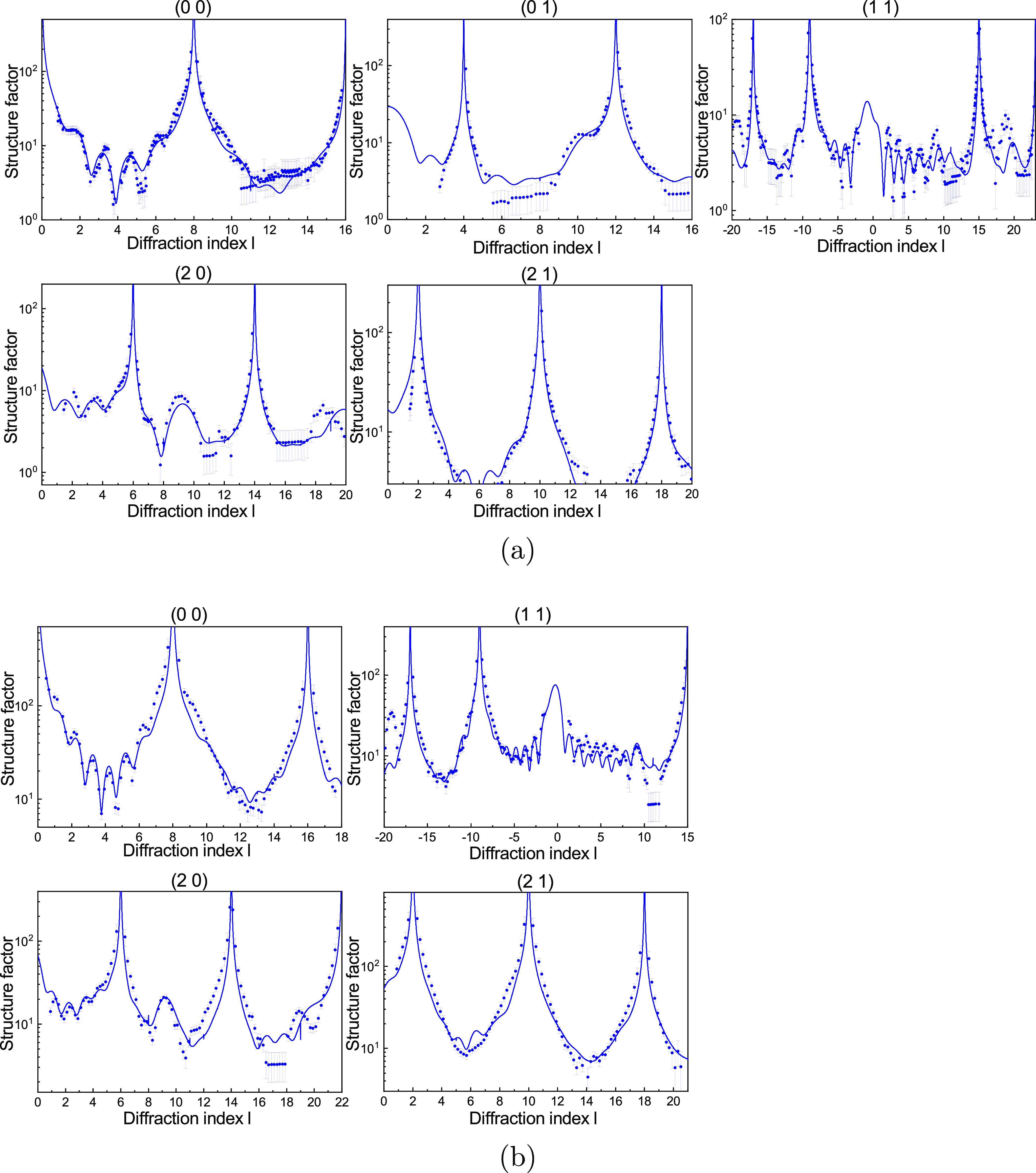
Experimental crystal truncation rods for calcite in contact with 10 mM (a) lauric acid and (b) stearic acid in methanol (symbols with error bars). The best model fits for each case, as described in the text, are shown as solid lines.

SXRD data sets were obtained for hexanoic acid (C6), octanoic acid (C8), lauric acid (C12), and stearic acid (C18) dissolved in methanol; the number of measured rods differed per system in order to optimize the information obtained within the limited synchrotron beam time. For calcite in contact with lauric acid and stearic acid, the crystal truncation rods are shown in Figure 1. The measured structure factors (blue symbols with error bars) show strong oscillations as a function of diffraction index . Such oscillations are not present in the bulk terminated calcite structure or the calcite–water structure^24^ and indicate the existence of a layer with a well-defined thickness around 10–20 Å. It is known that ethanol can order on the calcite surface^31^ and methanol could in principle have similar behavior, but this small molecule cannot form a layer of the observed thickness. The intensity oscillations we observe were noted before for the specular [or (0 0)] rod for stearic acid,^16^ but here we find that the oscillations also occur in the nonspecular rods for both lauric and stearic acid. This means that these two carboxylic acids molecules are adsorbed to the calcite surface and form a well-defined layer in which the molecules are also laterally well-ordered with respect to the calcite lattice. This is thus found to be energetically more favorable than methanol (or water) adsorption.

#### Interfacial Structure

A model of the interface was constructed to reproduce the measured structure factors. Following Fenter and Sturchio, a monolayer of carboxylic acid molecules is expected, but the lateral positions of the atoms are unknown.^16^ It has been suggested that at the surface, a calcium carboxylic acid bicarbonate is formed, a process in which one CO_2_ per Ca is removed.^4^ We, however, do not find evidence for any significant rearrangement or removal of atoms at the interface; thus, the calcite surface closely retains the structure it has in an aqueous environment.^24^ Many different models were tried; the model described here gave the best fit that is shown as the blue curves in Figure 1. We previously found small deviations from the bulk positions of calcite for the topmost CaCO_3_ layer and negligible deviations for the second CaCO_3_ layer.^24^ The interfacial model is thus constructed starting with the bulk atomic positions, in which displacements were only allowed in the top CaCO_3_ layer. The CO_3_^2–^ moiety was considered as a group. Fitting parameters for Ca^2+^ and CO_3_^2–^ were displacements in the *x*, *y*, and *z* directions and an isotropic Debye–Waller parameter. The CO_3_^2–^ moiety was also allowed to rotate in the *x*, *y*, and *z* directions. All displacements in the calcite crystal were constrained to fulfill the glide plane symmetry of calcite. On top of the calcite crystal, two independent carboxylic acid molecules per unit cell were added with positions and tilts as fitting parameters. For simplicity, we used rigid molecules since this already yields satisfactory fits from which the important structural features of the system can be derived. Since the carboxylic acid molecules are quite long, a decrease in order for increasing distance from the interface is expected. To model this and limit the total number of fitting parameters, four to six adjacent carbon molecules in the fatty acid molecule are given the same Debye–Waller parameter, resulting in a total of four Debye–Waller parameters for lauric acid and five for stearic acid. The two O atoms of the fatty acid were given a single Debye–Waller parameter. On top of the carboxylic acid molecules, isotropic liquid was added representing the unordered methanol solvent.

**Figure 2:**
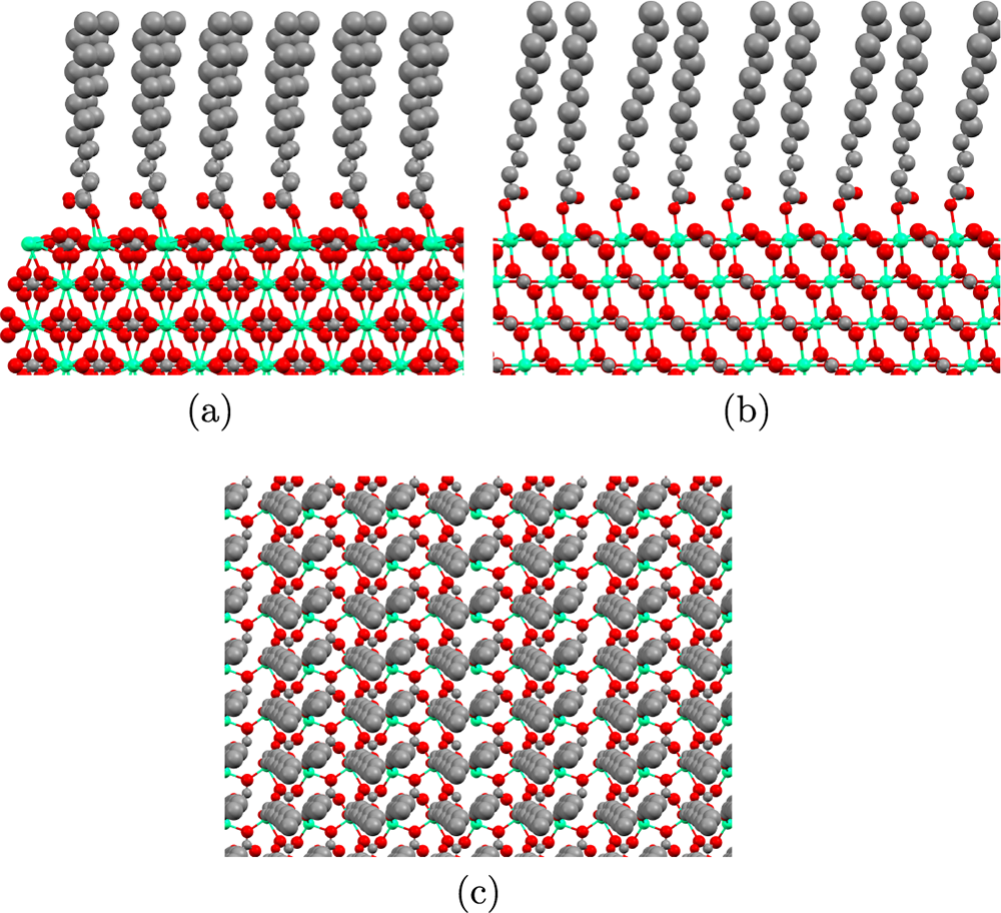
View of the calcite–lauric acid interface along the (a) *bc* plane, (b) *ac* plane, and (c) *ab* plane. Carbon, oxygen, and calcium are depicted in gray, red, and green respectively. The size of the atoms denotes their thermal disorder, and this increases in the carboxylic acid molecules for increasing distance from the surface.

The final model of the calcite–lauric acid interface is shown in Figure 2, whereas that of the calcite–stearic acid interface can be found in Supporting Information S2. At the surface, the six-fold bonding of Ca^2+^ in the bulk crystal is broken. Similarly, the topmost O atoms of the carbonate moieties are undercoordinated. This is compensated for by the adsorption of the carboxylic acid. The O atom of the carboxylic acid, with a Ca–O distance of 2.6–2.7 Å, completes the surrounding as found in the bulk structure. The second O atom of the acid molecule compensates for the broken surface bonds of the O atoms of the topmost carbonate group. This O atom is found above the highest O atom of the carbonate group, at a distance of 2.5–2.9 Å. When calcite is in contact with water, the O atoms of the water molecules are found at nearly identical positions above the crystal.^24−33^ In all these cases, the calcite structure thus prefers adsorption at those two positions. This can therefore explain why adsorption of carboxylic acid is more favorable than other groups, such as alcohols.^14^ Rotations of the carbonate group in the top CaCO_3_ layer are small (<10°), and the atomic positions in this layer are found close to the bulk positions. Only the Ca^2+^ atom is shifted in the *b* direction, potentially to better accommodate the Ca–O bond with the carboxylic acid molecules.

When looking at the carboxylic acid layer, a full monolayer is expected at the concentrations that were used here.^16,4^ Indeed for lauric acid and stearic acid, we find an occupancy close to 100% (or 4.95 × 10^14^ molecules/cm^2^), which was fixed to 100% in the final model. This means that for each Ca^2+^ atom at the surface, there is one carboxylic acid molecule. The stearic acid monolayer itself has a height of 21.6–22.0 Å̊ from lowest O to the terminal C atom, which agrees with earlier measurements.^16^ The height of the lauric acid monolayer is 13.9–14.1 Å. This is slightly smaller than the length of the molecules because of the tilt of the molecules with respect to a fully vertical position. For both lauric acid and stearic acid, one of the two inequivalent molecules is tilted approximately 3° from an upright position, whereas the other molecule deviates approximately by 10° in the *a* direction. In the *b* direction, an angle of about 3° is found for both stearic acid molecules. For lauric acid, angles of 1 and 8° are found. This is summarized in Supporting Information S3, whereas the atomic positions of both carboxylic acid molecules can also be found in Supporting Information S4. In principle, the tilts of the fatty acid molecules can be in two directions, but then the glide plane symmetry of the surface is broken. This was incorporated in the model as two domains with equal occupancies, which did result in a better fit than a single domain fit. The Debye–Waller parameters of the organic molecules, a measure for the disorder of the atoms, increase for atoms further away from the surface (Figure 2). This increase is limited; the atoms still occupy well-defined positions (see Supporting Information S4 for the values).

### Hexanoic Acid and Octanoic Acid on Calcite

**Figure 3:**
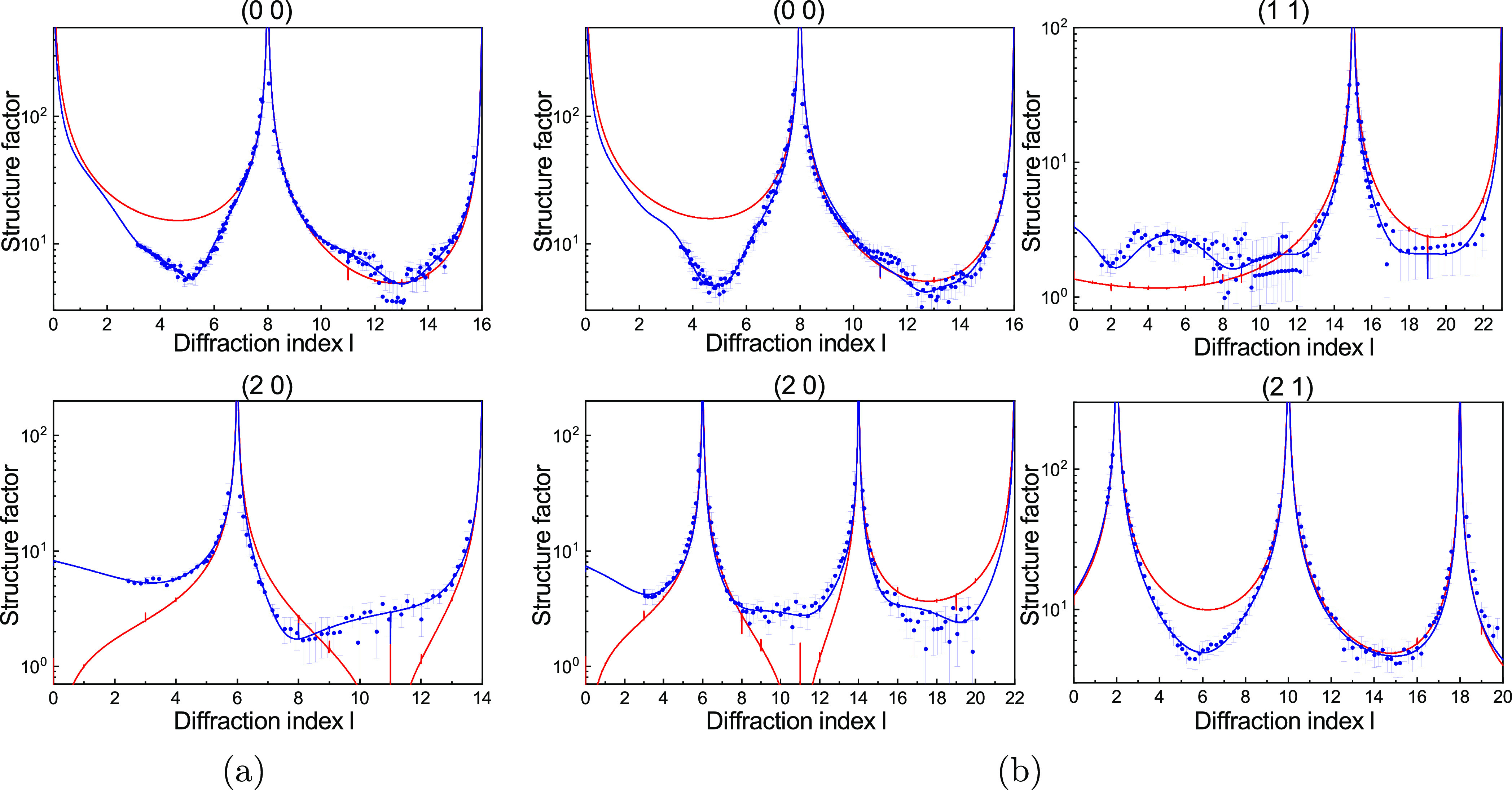
Experimental crystal truncation rods for calcite in contact with 10 mM (a) hexanoic acid and (b) octanoic acid in methanol (symbols with error bars). The best model fits for each condition, as described in the text, are shown as solid lines. The red solid line shows the calcite bulk-terminated structure without liquid.

In contrast to the long chain carboxylic acid molecules, the crystal truncation rods measured for octanoic acid and hexanoic acid (Figure 3) do not show oscillations, thus pointing toward a less ordered layer. Using X-ray reflectivity, Hakim et al. observed weak oscillations earlier for calcite in contact with a thin layer of octanoic acid,^15^ but in our experiments, these are not visible. This is probably caused by differences in the experimental conditions. In our experiments, a 10 mM carboxylic acid solution in methanol was used instead of the pure carboxylic acids as the liquid phase. Moreover, Hakim et al. used a thinner liquid layer than in our experiments, which can induce a more laterally ordered structure as was for example observed in water layers on NaCl.^34^

The measured crystal truncation rods for hexanoic acid and octanoic acid are similar. They clearly deviate from the calcite bulk-terminated structure without solution, shown as the red line in Figure 3. When looking at the specular rod in more detail, we observe a sharp minimum in the structure factors around a value of 5 for diffraction index  in both cases, which is not present in the calcite–water system.^24,35^ Besides this, there is strong experimental^15^ and theoretical evidence^14,36−38^ for the adsorption of short-chain carboxylic acids on the (104) calcite surface. The nonspecular crystal truncation rods look more similar to those of the calcite–water system,^24^ which is an indication of laterally disordered carboxylic acid molecules.

To fit the data, also here a model of the interface was constructed. At first, a model with two independent molecules was tested, similar to the models of lauric acid and stearic acid. This did not give a satisfactory fit, as oscillations were visible in the fitted rods. If the adsorbing molecules are fully disordered, it is possible to describe them with an isotropic liquid layer. With only this liquid layer, however, essential features in the crystal truncation rods could not be fitted. The fit in Figure 3 was achieved with a model that included the ordered carboxylic acid group (COO), an unordered layer of electron density representing the carbon chain, and an isotropic liquid representing methanol. Mainly, the O atoms were found at well-defined positions, which were similar to those of the long chain carboxylic acids (see Supporting Information S3 and S4). The C atom that is the first atom of the carbon chain is already found to have a large Debye–Waller parameter. The other C atoms in the chain are fully disordered, leading to a diffuse interface with the solution. This also explains why there is little difference between the measured rods of hexanoic acid and octanoic acid. As the chains are completely disordered, they only contribute to the (0 0) rod. The ordered COO atoms bind in a similar fashion for hexanoic acid and octanoic acid and therefore result in similar structure factors.

**Figure 4:**
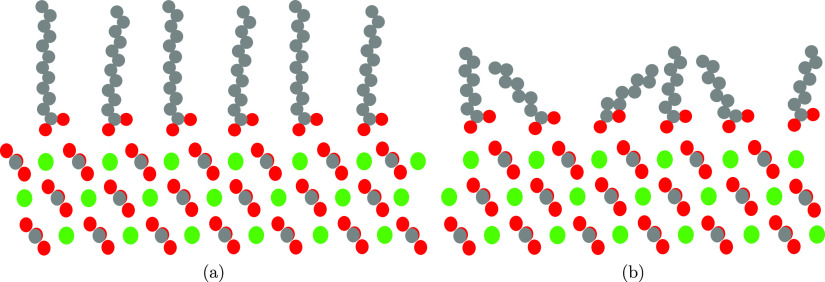
Schematic of (a) lauric acid adsorbing to calcite as an example of an ordered long-chain carboxylic acid and (b) octanoic acid adsorbing to calcite as an example of a partly disordered short-chain carboxylic acid. Carbon, oxygen, and calcium are depicted in gray, red, and green, respectively.

We thus find that both short- and long-chain carboxylic acid molecules adsorb at the calcite surface with the same adsorption site, but with very different ordering, as illustrated in Figure 4. The interactions of the long chains are stronger than for the short chains, and this leads to the fully ordered layers of lauric acid and stearic acid. In the short-chain hexanoic acid and octanoic acid layers, the entropy apparently dominates the free energy and the chains are disordered. These monolayers are in a very different environment than the bulk carboxylic acids, but we nevertheless find that the order corresponds to that in the bulk, i.e., solid for the long-chain and liquid for the short-chain molecules. The difference in ordering agrees with the results of Osman and Suter, who used NMR and IR spectroscopy and found solid-like behavior for longer fatty acids (≥C_10_) and disorder for shorter chains (≤C_10_).^4^

The formation of ordered monolayers of carboxylic acids and related molecules has been extensively studied for a variety of substrates.^39^ Such self-assembled monolayers (SAMs) are a way to functionalize a surface, but little has been reported on the formation of carboxylic acid SAMs on calcite. The inverse process, i.e., the formation of calcite on a SAM of functionalized carboxylic acid, has been shown to allow the control of calcite crystallization.^40,41^ We have here thus demonstrated the formation of highly ordered SAMs of lauric acid and stearic acid on calcite, while the SAMs of hexanoic acid and octanoic acid are much less ordered.

### Stearic Acid Adsorption on Muscovite Mica

In addition to calcite, we have also investigated the adsorption of stearic acid in methanol at a muscovite mica substrate, resembling a sandstone reservoir. Muscovite mica contains predominantly K^+^ surface ions (K-mica). To make it comparable with calcite, the K^+^ surface ions were exchanged for Ca^2+^ (Ca-mica).^42^ Both K-mica and Ca-mica in contact with a 10 mM stearic acid solution in methanol (Supporting Information S5) show diffraction patterns similar to those of K or Ca-terminated mica in aqueous solution (without stearic acid) and do not exhibit any oscillations of the type observed for calcite. Therefore, these experiments show that stearic acid does not adsorb at muscovite mica under these conditions. Molecular dynamics simulations suggested that Ca^2+^ could bridge between muscovite and deprotonated decanoic acid (i.e., decanoate).^43^ These simulations were performed in an aqueous environment, which is experimentally not possible due to the low solubility of stearic acid in water. Nevertheless, the simulations show that binding does not take place when the carboxylic acid molecule is protonated. The bridging effect only takes place when the molecule is deprotonated. In our experiments in methanol, stearic acid is most likely not deprotonated and thus does not adsorb. As stearic acid has the longest carbon chain of the carboxylic acid molecules used here, we do not expect that shorter carboxylic acids adsorb at the muscovite mica surface. Under different experimental conditions than used here, the bridging role of Ca^2+^ is possible, for example, at the mineral–oil interface in the presence of reservoir water, which allows for deprotonation. This has been demonstrated for muscovite surfaces in contact with decane containing stearic acid,^44,45^ which shows that cation bridging probably plays an important role in the low-salinity effect in enhanced oil recovery.^46,47^

### Calcite versus Muscovite Mica

This leaves us with the question why stearic acid adsorbs at a calcite substrate but does not at a Ca–mica substrate. After exchange, a surface Ca^2+^ coverage close to 25% is expected on muscovite.^42,48^ This corresponds to approximately 1.07 × 10^14^ Ca^2+^ ions/cm^2^, which is nearly five times less than 4.95 × 10^14^ Ca^2+^ ions/cm^2^ in calcite and thus, assuming each carboxylic acid molecule binds to a Ca^2+^ ion, the gain in enthalpy due to the interaction of neighboring molecules is much reduced. Moreover, adsorption at the calcite surface completes the surrounding of Ca^2+^ and the topmost O atom and in this way compensates for the unfavorable dangling bonds at the calcite surface. The adsorbed carboxylic acid molecule binds simultaneously to Ca and O atoms. For muscovite mica, the situation is very different because there ionic interactions play a large role in bonding. Apparently, the interaction muscovite Ca^2+^ has with the protonated carboxylic acid group is not strong enough to adsorb. Hydrophobic interactions between the carbon chains of the carboxylic acid molecules increase with longer chains but are expected to be of similar strength for both substrates. Whereas the carboxylic acid group can provide oxygen atoms at locations that resemble the crystal structure of calcite, this is not the case for muscovite. The structure of muscovite mica consists of aluminosilicate sheets. They contain a negative charge, which is compensated for by layers with cations. The charge-based interactions between the layers are relatively weak, as shown by the ease of cleaving them. Within the layers, the atoms are covalently bound, but the adsorbing molecules do not have the required charge to bind to the Ca–mica surface.

## Conclusions

Surface X-ray diffraction was used to determine the adsorption structure of carboxylic acids on calcite. The long chain carboxylic acid molecules lauric acid and stearic acid showed clear oscillations in the measured crystal truncation rods. This means that monolayers of carboxylic acid molecules are formed that are strongly ordered in both in-plane and out-of-plane directions and can be considered as examples of well-ordered self-assembled monolayers on this substrate. The carboxylic acid molecules are aligned almost vertically with respect to the calcite surface, with a maximum tilt of 10°. Adsorption of short-chain carboxylic acid molecules hexanoic acid and octanoic acid was also confirmed using diffraction. Whereas the carboxylic acid group close to the surface possesses some order, the carbon chain is fully disordered for both molecules. The O atoms of the carboxylic acid molecules bind at similar positions to water molecules at the calcite–water interface. This suggests that these positions are prone to adsorption of oxygen-bearing organic molecules. The adsorption of stearic acid on calcite was compared to that on K^+^ and Ca^2+^-functionalized muscovite mica. We find no evidence for adsorption at the muscovite (001) surface, which is explained by the lack of ionic interactions with the neutral carboxylic acid molecules.

While natural oil reservoirs have a far more complex composition than the simple systems studied here, our results do show the main differences in the bonding mechanisms of protonated carboxylic acids between calcite and muscovite mica, as model systems for carbonate and sandstone reservoirs, respectively. Our 3D structure determinations should also be useful for comparison with atomic-scale computer simulations.

## Supporting Information Available

Experimental set-up; details and parameters of interface structures; crystal truncation rod data on muscovite mica (PDF)
