# Supplementary material for: Ordered and Disordered Carboxylic Acid Monolayers on Calcite (104) and Muscovite (001) Surfaces
Source: J Phys Chem C Nanomater Interfaces. 2022 May 18;126(20):8855–62. doi: 10.1021/acs.jpcc.2c01157 (PMC9150091; doi:10.1021/acs.jpcc.2c01157)
Supplement: Supplementary file 1 — jp2c01157_si_001.pdf [file jp2c01157_si_001.pdf]

Ordered and Disordered Carboxylic Acid Monolayers on Calcite (104) and Muscovite (001) Surfaces

Sander J.T. Brugman, Paolo Accordini, Frank Megens, Jan-Joris Devogelaer, and Elias Vlieg*

Radboud University, Institute for Molecules and Materials, Heyendaalseweg 135, 6525AJ Nijmegen, The Netherlands

E-mail: e.vlieg@science.ru.nl

S1: Experimental set-up

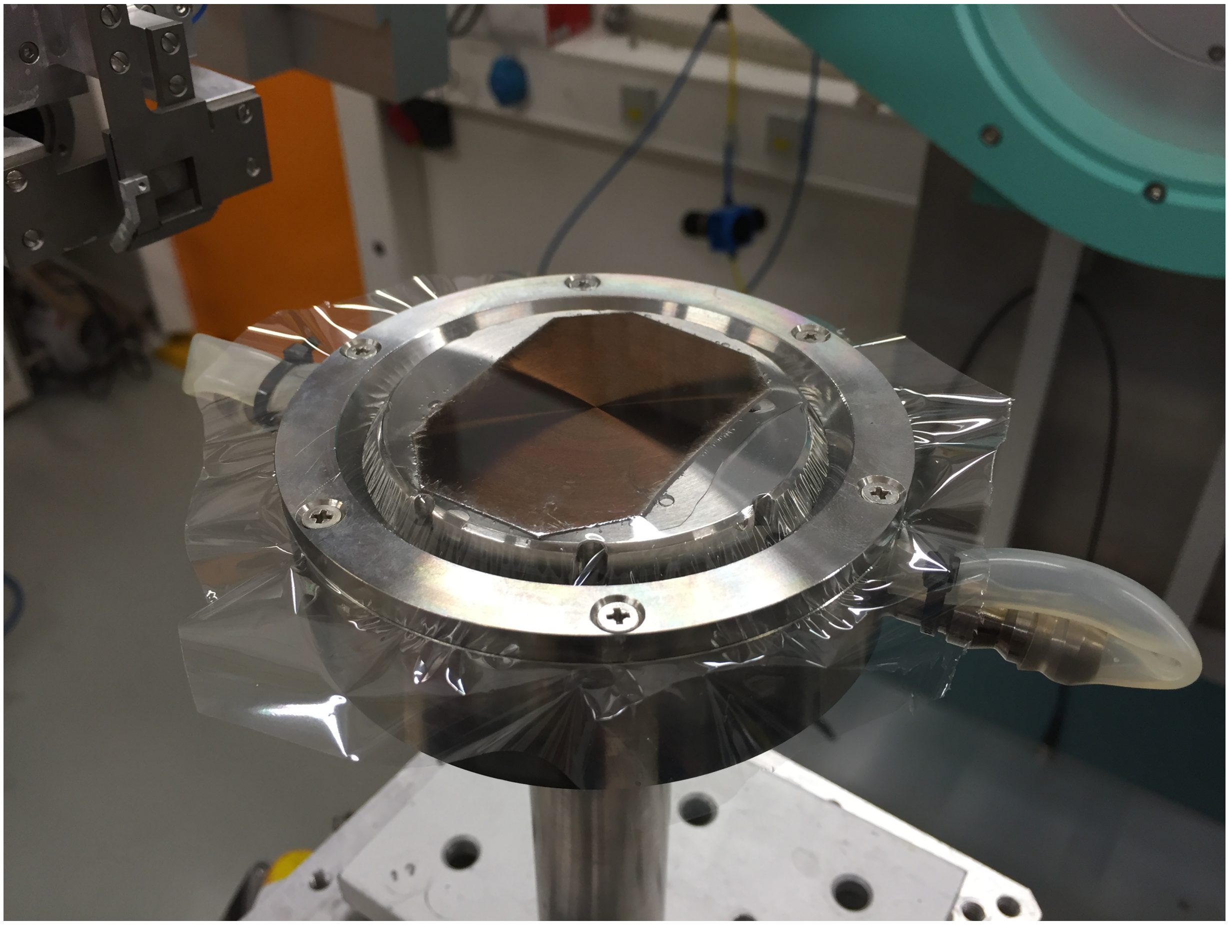

Figure S1: A photograph of the experimental set-up mounted on the X-ray diffractometer. The stainless steel cell has a plateau for mounting the crystal and a ring with an O-ring seal that allows the positioning of a mylar foil. In this case a muscovite mica crystal is mounted and the excess solution is visible. X-rays enter and leave from the top.

S2: Calcite - stearic acid interface

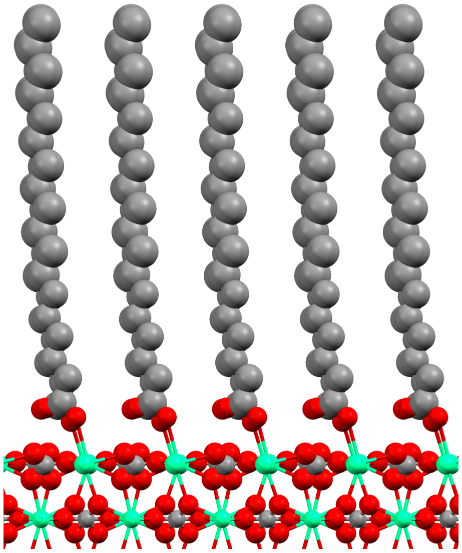

(a)

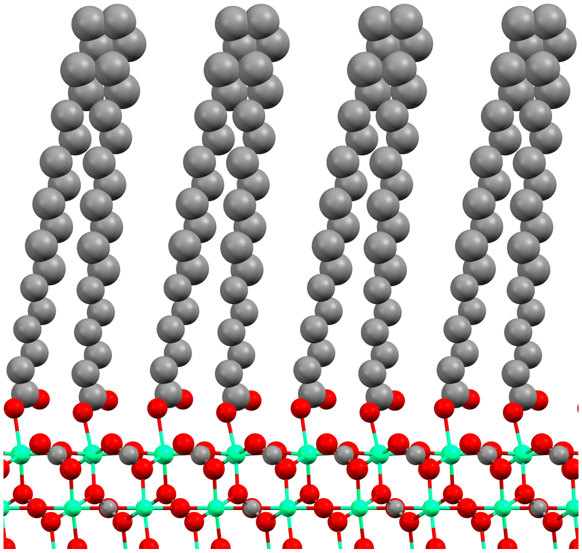

(b)

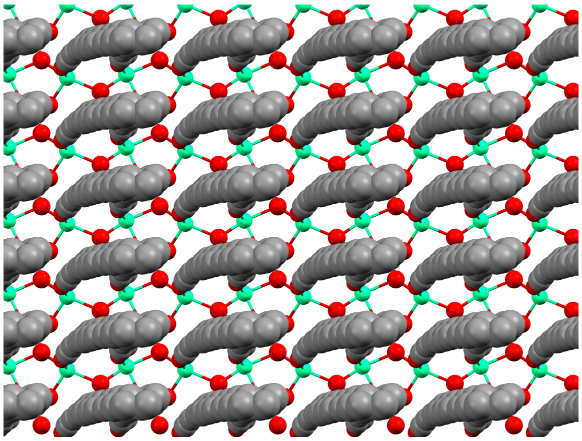

(c)

Figure S2: View of the calcite-stearic acid interface along the (a) *bc*-plane (b) *ac*-plane and (c) *ab*-plane. Carbon, oxygen and calcium are depicted in grey, red and green respectively.

S3: Structural parameters

Table S1: Structural parameters of the carboxylic acid molecules adsorbed at calcite, derived from the best fit model. The height of the bulk Ca^{2+} atoms of the top layer is defined as 0 in the z-direction.

Compound	χ^2	molecule	α angle (°)	β angle (°)	height$_{start}$ (Å)	height$_{molecule}$ (Å)
Lauric acid	2.9	1	92	94	2.3	14.1
		2	85	100	2.5	13.9
Stearic acid	6.9	1	94	95	2.4	22.0
		2	95	101	2.6	21.7
Octanoic acid	0.7	1	flexible	flexible	2.0	flexible
		2	flexible	flexible	2.5	flexible
Hexanoic acid	1.0	1	flexible	flexible	2.4	flexible
		2	flexible	flexible	2.5	flexible

S4: Atomic positions

Table S2: Atomic positions parameters of hexanoic acid and octanoic acid adsorbed at calcite, derived from the best fit model. The height of the bulk Ca^{2+} atoms of the top layer is defined as 0 in the z-direction. Atoms of the carboxylic acid molecules are numbered from the mineral surface. The position of surface Ca^{2+} is given for reference.

Hexanoic acid	Molecule 1			Molecule 2			
Atom	x (Å)	y (Å)	z (Å)	x (Å)	y (Å)	z (Å)	Debye-Waller (Å)
Ca	4.75	2.46	-0.07	0.70	2.53	-0.07	0.13
O_1	4.43	3.16	2.43	1.05	4.80	2.54	0.09
O_2	5.79	4.70	3.38	7.88	2.92	2.72	0.09
C_1	5.03	3.69	3.41	0.44	3.90	3.20	0.41
Octanoic acid	Molecule 1			Molecule 2			
Atom	x (Å)	y (Å)	z (Å)	x (Å)	y (Å)	z (Å)	Debye-Waller (Å)
Ca	0.86	2.67	-0.05	4.91	2.32	-0.05	0.15
O_1	0.64	3.22	2.04	3.88	3.09	2.46	0.09
O_2	2.10	4.42	3.30	5.10	0.01	2.65	0.09
C_1	1.25	3.50	3.12	4.49	3.99	3.12	0.42

Table S3: Atomic positions parameters of lauric acid and stearic acid adsorbed at calcite, derived from the best fit model. The height of the bulk Ca^{2+} atoms of the top layer is defined as 0 in the z-direction. Atoms of the carboxylic acid molecules are numbered from the mineral surface. The position of surface Ca^{2+} is given for reference.

Lauric acid	Molecule 1			Molecule 2			
Atom	x (Å)	y (Å)	z (Å)	x (Å)	y (Å)	z (Å)	Debye-Waller (Å)
Ca	4.79	2.21	-0.04	0.75	2.77	-0.04	0.13
O_1	4.54	2.93	2.26	0.34	3.09	2.52	0.09
O_2	5.66	4.75	3.03	1.58	4.79	3.38	0.09
C_1	4.99	3.68	3.18	0.89	3.72	3.48	0.16
C_2	4.69	3.21	4.47	0.71	3.11	4.72	0.16
C_3	5.25	4.07	5.57	1.41	3.81	5.85	0.16
C_4	4.82	3.51	6.83	1.09	3.13	7.08	0.16
C_5	5.44	4.23	7.98	1.85	3.68	8.23	0.28
C_6	4.99	3.62	9.21	1.50	2.95	9.42	0.28
C_7	5.64	4.25	10.39	2.29	3.42	10.60	0.28
C_8	5.20	3.60	11.60	1.95	2.64	11.76	0.28
C_9	5.87	4.19	12.80	2.75	3.07	12.94	0.36
C_{10}	5.43	3.54	14.00	2.41	2.29	14.10	0.36
C_{11}	6.12	4.12	15.19	3.23	2.71	15.27	0.36
C_{12}	5.65	3.48	16.38	2.87	1.94	16.42	0.36
Stearic acid	Molecule 1			Molecule 2			
Atom	x (Å)	y (Å)	z (Å)	x (Å)	y (Å)	z (Å)	Debye-Waller (Å)
Ca	0.82	2.60	-0.01	4.87	2.39	-0.01	0.13
O_1	0.28	3.32	2.36	4.49	3.07	2.59	0.14
O_2	1.70	4.97	3.03	5.93	4.75	3.09	0.14
C_1	0.84	4.06	3.23	5.12	3.82	3.40	0.26
C_2	0.43	3.75	4.54	4.86	3.52	4.74	0.26
C_3	1.13	4.57	5.60	5.65	4.36	5.71	0.26
C_4	0.57	4.21	6.88	5.24	3.99	7.04	0.26
C_5	1.30	4.89	7.99	6.06	4.69	8.08	0.26
C_6	0.72	4.48	9.25	5.63	4.28	9.38	0.26
C_7	1.45	0.07	10.40	6.47	4.89	10.45	0.36
C_8	0.87	4.60	11.64	6.04	4.43	11.75	0.36
C_9	1.62	0.17	12.81	6.89	0.02	12.83	0.36
C_{10}	1.04	4.70	14.04	6.46	4.54	14.11	0.36
C_{11}	1.80	0.24	15.21	7.33	0.11	15.19	0.36
C_{12}	1.20	4.78	16.43	6.87	4.65	16.47	0.36
C_{13}	2.13	0.32	17.85	7.93	0.22	17.78	0.36
C_{14}	1.55	4.85	19.07	7.50	4.74	19.06	0.36
C_{15}	2.48	0.39	20.49	0.47	0.31	20.37	0.45
C_{16}	1.91	4.92	21.72	0.03	4.84	21.65	0.45
C_{17}	2.84	0.46	23.14	1.10	0.41	22.96	0.45
C_{18}	2.26	0.00	24.36	0.67	4.93	24.25	0.45

S5: Crystal truncation rods muscovite mica

The data of K^{+}-functionalised or Ca^{2+}-functionalised muscovite mica in solutions of stearic acid in methanol is very similar to previously published data of Ca^{2+}-terminated muscovite mica in aqueous solution,[1] demonstrating that no stearic acid adsorption takes place.

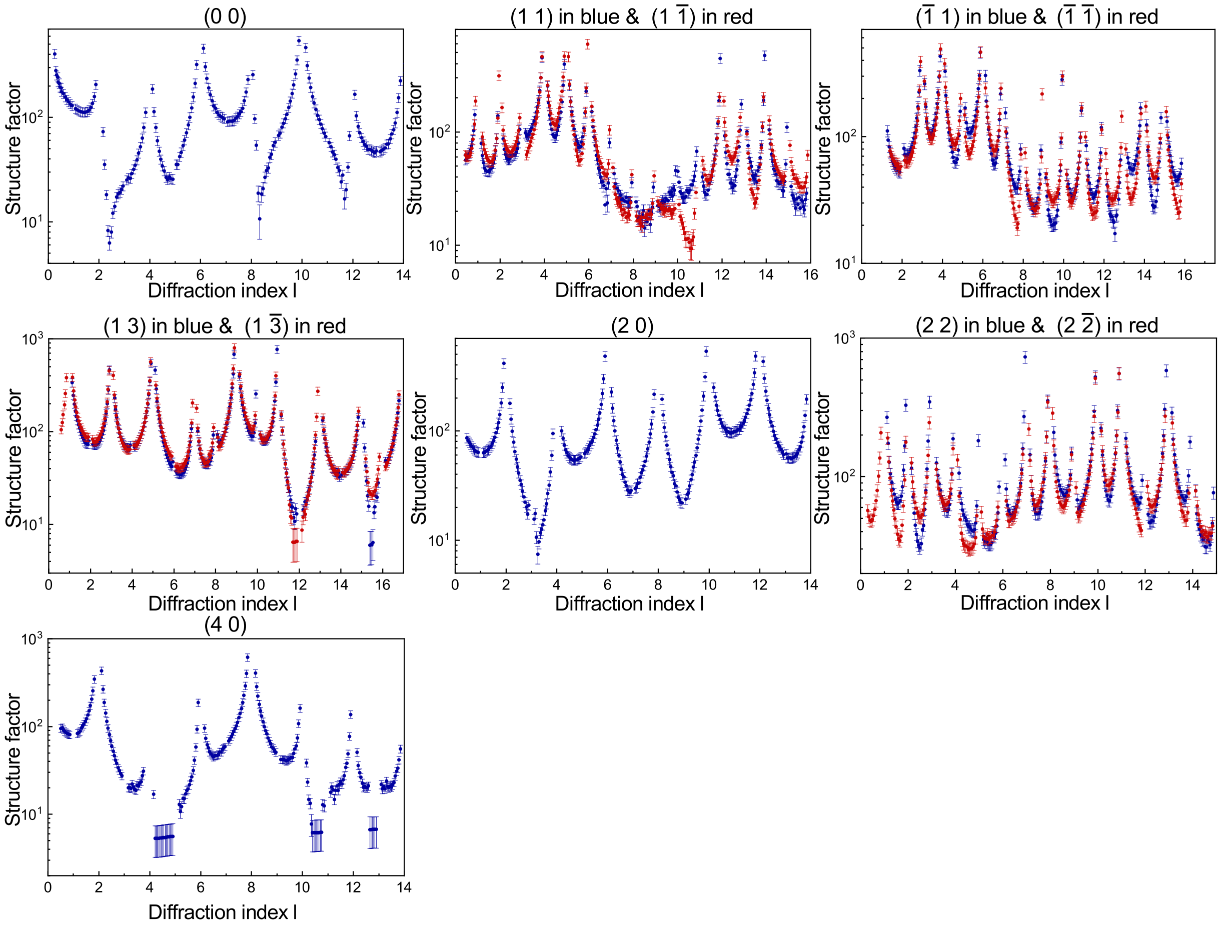

Figure S3: Experimental specular and ten unique non-specular rods (blue and red symbols with error bars) for K^{+}-functionalised muscovite mica in contact with a 10 mM solution of stearic acid in methanol.

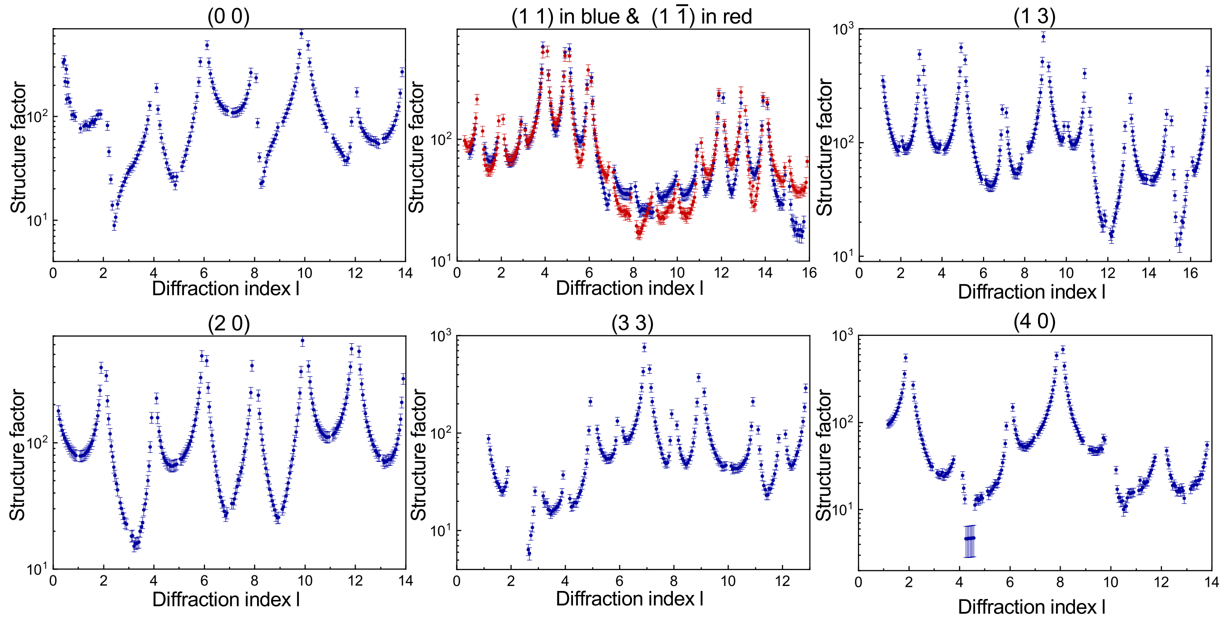

Figure S4: Experimental specular and six unique non-specular rods (blue and red symbols with error bars) for Ca^{2+}-functionalised muscovite mica in contact with a 10 mM solution of stearic acid in methanol.

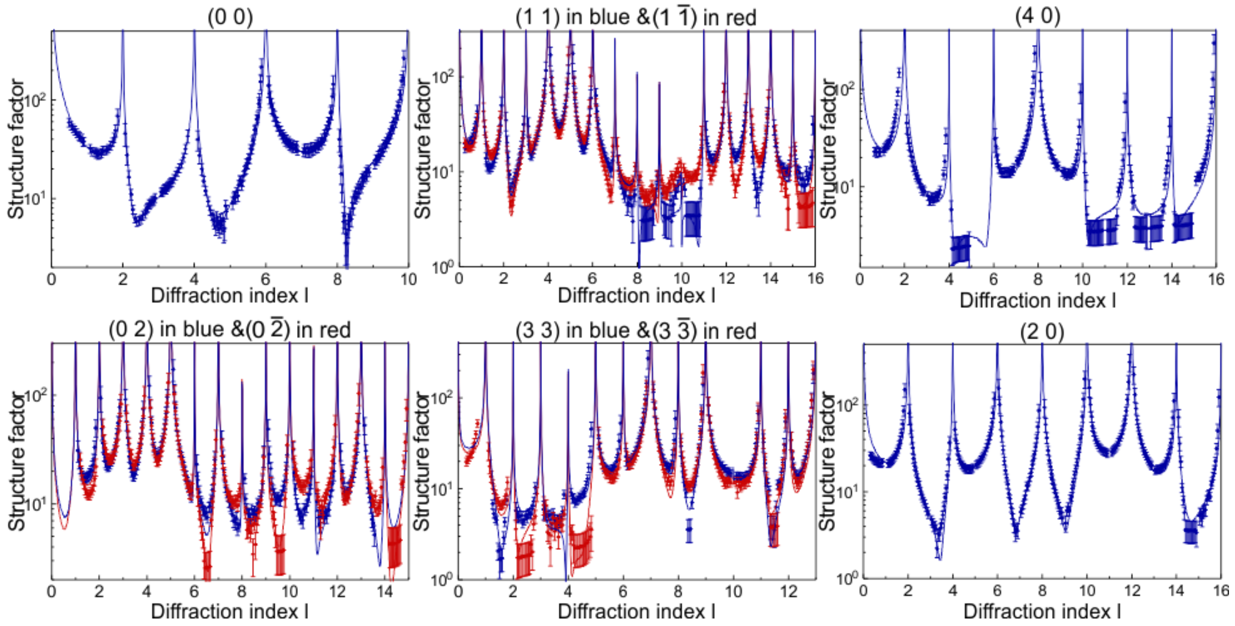

Figure S5: Experimental specular and nine non-specular rods (blue and red symbols with error bars) for muscovite mica in contact with a 25 mM $CaCl_2$ aqueous solution. A model fit is shows as a solid line. Figure adapted from the Supporting Information from Brugman et al.[1]

References

(1) Brugman, S. J. T.; Werkhoven, B. L.; Townsend, E. R.; Accordini, P.; van Roij, R.; Vlieg, E. Monovalent-divalent cation competition at the muscovite mica surface: experiment and theory. *J. Coll. Interf. Sci.* **2020**, *559*, 291–303.
